# Double Uterus

**Published:** 1914-08

**Authors:** 


**Double Uterus.** W. L. Duffield, L. I. Med. Jour., July, 1914, describes a case in a negro girl aged 20 who had borne one child previously, having sustained a laceration of the perineum with cystocele and proctocele, and so extensive a laceration of the cervix that it was impossible to determine the question of a double cervical canal. On operation a right pyosalpinx, much adherent was removed, the bowel being ruptured during the operation but sutured so that no unfavorable complication occurred. The corresponding uterus was left in place. There was then found a mass which was removed and which proved to be a left pregnant uterus. The right pyosalpinx was probably due to a septic abortion.

